# Enumeration of organisms occurring in the intestinal tract and yolk sac of day-old chicks from commercial hatcheries

**DOI:** 10.1007/s11259-026-11447-2

**Published:** 2026-08-05

**Authors:** Karely Cantu, Craig Coufal, Michael Rothrock, Steven C. Ricke

**Affiliations:** 1 Poultry Science Dept, Texas A&M University, College Station, TX 77843 USA; 2 United States Department of Agriculture, Agricultural Research Service, Athens, GA 30605 USA; 3 Department of Animal and Dairy Sciences, University of Wisconsin - Madison, Office 2124 MSABD, 1933 Observatory Drive, Madison, WI 53706 USA

**Keywords:** Chicks, Commercial, Hatcheries, Intestines, Yolk sac, Microorganisms, Yeast, Molds

## Abstract

This study evaluated the naturally occurring microorganisms within the gastrointestinal tract (GIT) and yolk sacs of day-old commercial broiler chicks. Across 6 independent trials, day-old chicks (*n* = 10 per trial) were obtained from various commercial hatcheries, humanely euthanized, and necropsied. The entire intestine (duodenum to colon) and the yolk sac were isolated, homogenized, serially diluted, and plated onto selective media to enumerate total aerobes, *Clostridium* spp., *Clostridium perfringens*,* Staphylococcus aureus*, Gram-negative bacteria, *Escherichia coli*, total coliforms, and yeast and molds. Intestinal microbial loads were consistently high, with counts for both total aerobes and *Clostridium* spp. (8.14 and 8.11 log_10_ cfu/mL, respectively). High counts for intestinal *S. aureus*, Gram-negative bacteria, *E. coli*, and total coliforms were also observed (6.57, 7.44, 6.57, and 7.60 log_10_ cfu/mL, respectively). Intestinal yeast and mold counts averaged 5.49 log_10_ cfu/mL. Intestinal *C. perfringens* counts were uniformly low, averaging 2.06 log_10_ cfu/mL across all trials. In comparison, yolk sac microbial counts were lower, averaging 3.70, 3.67, and 1.52 log_10_ cfu/mL for total aerobes, *Clostridium* spp., and *C. perfringens*, respectively. These results demonstrate that commercial broiler chicks harbor dense aerobic and anaerobic intestinal populations immediately upon hatch, whereas the yolk sac maintains substantially lower microbial loads. The discrepancy suggests that colonization occurs via trans-shell migration of eggshell microorganisms during incubation. Conversely, the high intestinal microbial loads appear to be primarily driven by the ingestion of environmental microorganisms circulating within the commercial hatchery environment at the time of hatch.

## Introduction

Contamination of the maternal oviduct and egg yolk has been shown to contain microbiome taxa that were also detected in the embryo intestinal microbiome (Ding et al. 2022; Jin et al. 2022). In addition, environmental sources and eggshell microbiota can also contribute to gastrointestinal microbial consortia (Maki et al. 2020). Finally, intestinal microbial populations increase markedly during pipping and hatching of the chick (Meisinger et al. 2026b). The composition of this microbiota in newly hatched chicks is crucial, as early-life microbial successions exert long-term effects on bird health, immunological homeostasis, and grow-out performance during rearing (Maki et al. 2020).

The chicken gastrointestinal tract (GIT) microbiota continues to evolve during post-hatch upon exposure to external farm environments (Feye et al. 2020). In adult poultry, the mature GIT microbiota is populated by various microorganisms influenced by host genetics, sex, chronological age, dietary formulation, and environmental conditions (Feye et al. 2020). Consequently, the baseline composition of the “normal” intestinal microbiota of broiler chickens may vary between commercial flocks. The upper and lower intestinal segments (duodenum, jejunum, and ileum) are predominantly colonized by *Lactobacillus*,* Streptococcus*,* Enterococcus*, and *Clostridium* spp. (Feye et al. 2020). Conversely, the cecal pouches harbor an obligate anaerobic community predominantly populated by *Clostridium* spp. and other firmicutes as well as more minor organisms (Feye et al. 2020).

Characterizing early GIT colonization dynamics is important because the microbiota directly regulates the functional development of intestinal mucosa. Early symbionts modulate nutrient transport, protein and carbohydrate fermentation, mucosal barrier integrity, and the maturation of the gut-associated lymphoid tissue (GALT) (Dittoe et al. 2022). Despite its biological significance, few studies have quantified the viable microbial loads that reside in the intestine and yolk sac of newly hatched chicks.Therefore, the objective of this study was to determine the indigenous microorganisms in the intestine and yolk sac of day-old chicks obtained from commercial hatcheries.

## Materials and methods

### Chicks and euthanasia

Six trials were conducted using day-old broiler chicks obtained from various commercial hatcheries. Chicks selected for sampling were of high visual quality and free of deformities or external infections. After chicks were obtained from the various commercial hatcheries, they were transferred to Texas A&M University for euthanasia and necropsy. Animal handling and procedures conducted throughout the course of this study at Texas A&M University, College Station, TX were in accordance with Texas A&M University Institutional animal care and use committee (IACUC 2021-0056).

Chicks were euthanized in airtight bags using a high concentration of carbon dioxide (CO_2_) according to established animal welfare standards (Coenen et al. 2000; Leary 2013). Five chicks were euthanized per airtight bag for all trials. The chicks were supplemented CO_2_ gas until unconsciousness was achieved, and approximately 30 min passed prior to removal from the bag. After verification of death, chicks were immediately necropsied for intestinal and yolk sac microbial analysis.

### Microbial analysis

Ten chicks were sampled per trial (*n* = 60 total) for intestinal and yolk sac microbial enumeration. Prior to necropsy, the chicks were dipped into Nolvasan S disinfectant solution (Zoetis US, Parsippany, NJ) at a concentration recommended by the manufacturer. Tissue forceps and dissecting scissors were dipped in 100% ethanol followed by flaming for sterilization. Sterilized tools were used to necropsy and isolate the intestine and yolk sac of each chick per trial.

The intestinal sample collected per chick was composed of the duodenum, jejunum, ileum, ceca, and colon. The yolk sac was isolated and evaluated separately. Once isolated, the intestine and yolk sac were placed into sterile Whirl-pak bags (Nasco, Fort Atkinson, WI). Intestinal samples were cut into smaller portions inside the bag with sterile dissecting scissors to aid in homogenization.

The intestine and yolk sacs were weighed, and sterile phosphate-buffered saline (PBS, pH 7.4; HiMedia Laboratories, West Chester, PA) was added at a volume of 4 times their tissue weight, achieving a 1:5 ratio of sample weight to total volume. The contents were homogenized by massaging by hand vigorously by hand for approximately 1 min. Ten-fold serial dilutions were performed and plated onto various media.

The media used, microorganisms enumerated, plating volumes, and limits of detection (LOD) for each trial are listed in Table 1. Cultured colonies enumerated were calculated as log_10_ cfu/mL. The volume plated onto Petrifilms was 1.00 mL, and therefore the LOD was 5.00 cfu/mL (0.70 log_10_ cfu/mL). The volume plated for agars was 0.10 mL, and therefore, the LOD was 50 cfu/mL (1.70 log_10_ cfu/mL). Intestines and yolk sacs that had zero detectable counts were assigned a value of 0.40 log_10_ cfu/mL for Petrifilms and 1.40 log_10_ cfu/mL for agars for statistical analysis.

**Table 1:** Media used to enumerate various microorganisms in intestinal and yolk sac sampled from day-old chicks, the volume plated for each media and the calculated limit of detection (LOD) for 6 trials

Trial	Media	Microorganism	Volume plated (mL)	LOD (log_10_)
1 to 5	Tryptic Soy Agar (TSA)	Total aerobes	0.10	1.70
	*Staphylococcus* Express Count Petrifilm (STX)	*S. aureus*	1.00	0.70
	MacConkey Agar (MC)	Gram negative enteric bacilli	0.10	1.70
	Levine Eosin Methylene Blue Agar (EMB)	*E. coli* and total coliforms	0.10	1.70
	Yeast & Mold (YM) Petrifilm	Yeast and Mold	1.00	0.70
	Egg Yolk Agar (EYA)	*Clostridia* spp.	0.10	1.70
	Shahidi Ferguson Perfringens agar base (SFP)	*C. perfringens*	0.10	1.70
6	Aerobic Plate Counts (APC) Petrifilm	Total aerobes	1.00	0.70
	*Staphylococcus* Express Count Petrifilm (STX)	*S. aureus*	1.00	0.70
	MacConkey Agar (MC)	Gram negative enteric bacilli	0.10	1.70
	*E. coli* / coliform count Petrifilm (EC)	*E. coli* and total coliforms	1.00	0.70
	Yeast & Mold (YM) Petrifilm	Yeast and mold	1.00	0.70
	Egg Yolk Agar (EYA)	*Clostridia* spp.	0.10	1.70
	Shahidi Ferguson Perfringens agar base (SFP)	*C. perfringens*	0.10	1.70

### Statistical analysis

Data were analyzed using a one-way analysis of variance (ANOVA) using the model y=trial. Assumptions of equal variance and normality were met for ANOVA with means separated by LSD post hoc test. Data that did not meet ANOVA assumptions were analyzed using the Kruskal-Wallis test. Mean differences were considered significant at *P* < 0.05.

## Results

### Intestinal microbial counts

The results for intestinal microbial counts per trial and microorganism are shown on Table 2. The hatchery in Trial 6 had lowest microbial counts compared to the other hatcheries, with the exception of fungi and *C. perfringens*. Due to these LOD differences, Trial 6 was excluded from statistical analysis for total aerobes, *E. coli*, and coliforms (Table 1). Excluding Trial 6, all total aerobe intestinal counts were greater than 7.00 log_10_ cfu/mL for each hatchery with an overall average (Trials 1 through 5) of 8.14 log_10_ cfu/mL. The intestinal *Clostridium* spp. counts, excluding Trial 6, were also greater than 7.00 log_10_ cfu/mL for each hatchery with an overall average (Trials 1 through 5) of 8.11 log_10_ cfu/mL.

**Table 2:** Average intestinal microbial counts (log_10_ cfu/mL ± SE) per microorganism evaluated for Trials 1 through 6

Trial	Total aerobes^1^	S. aureus	Gram negatives	E. coli^1^	Coliforms^1^	Yeast and mold	Clostridium spp.	C. perfringens
1	7.70 ± 0.42^cd^	6.29 ± 0.47^bc^	6.90 ± 0.78^abcd^	6.15 ± 1.05^a^	6.97 ± 0.74^ab^	5.26 ± 0.07^b^	7.52 ± 0.37^b^	1.90 ± 0.26^b^
2	8.72 ± 0.09^a^	7.58 ± 0.12^ab^	8.23 ± 0.08^a^	6.95 ± 0.93^a^	8.53 ± 0.09^a^	4.92 ± 0.35^bc^	8.73 ± 0.05^a^	3.85 ± 0.49^a^
3	8.70 ± 0.07^ab^	7.30 ± 0.56^a^	7.87 ± 0.62^c^	6.74 ± 0.95^a^	7.97 ± 0.62^a^	5.34 ± 0.09^b^	8.70 ± 0.10^a^	1.49 ± 0.05^b^
4	7.14 ± 0.44^d^	4.58 ± 0.35^d^	6.31 ± 0.61^d^	5.48 ± 0.86^b^	6.42 ± 0.69^b^	4.40 ± 0.12^c^	7.33 ± 0.42^b^	1.60 ± 0.21^b^
5	8.43 ± 0.13^bc^	7.07 ± 0.12^c^	7.88 ± 0.07^b^	7.53 ± 0.57^a^	8.10 ± 0.04^b^	8.03 ± 0.08^a^	8.29 ± 0.15^b^	1.64 ± 0.17^b^
6	4.72 ± 0.73	2.50 ± 0.59^e^	4.61 ± 0.58^d^	3.73 ± 0.85	3.73 ± 0.85	4.98 ± 0.56^bc^	4.87 ± 0.56^c^	1.91 ± 0.18^b^
Avg 1–5	8.14 ± 0.31	6.57 ± 0.54	7.44 ± 0.36	6.57 ± 0.35	7.60 ± 0.39	5.59 ± 0.63	8.11 ± 0.29	2.10 ± 0.44
Avg1-6	7.57 ± 0.62	5.89 ± 0.81	6.95 ± 0.57	6.10 ± 0.55	6.95 ± 0.72	5.49 ± 0.53	7.57 ± 0.59	2.06 ± 0.36

^a−e^ Means within a column having different superscripts are significantly different (*P* < 0.05)

^1^ Total aerobes, *E.coli*, and coliform counts in Trial 6 were excluded from statistical analysis due to LOD differences from Trials 1 through 5

The intestinal results for *S. aureus*, Gram-negative bacteria, *E. coli*, and total coliforms were also high, excluding the hatchery from Trial 6 (6.57, 7.44, 6.57, and 7.60 log_10_ cfu/mL, respectively). However, the yeast and mold enumeration demonstrated that only one hatchery had high counts, resulting in a lower cumulative average than the aerobic microorganisms (5.49 log_10_ cfu/mL, including Trial 6). The hatchery from Trial 5 had the highest intestinal yeast and mold counts at 8.03 log_10_ cfu/mL (*P* < 0.01) compared to the other trials. Minimal intestinal *C. perfringens* were observed for all hatcheries, with the hatchery from Trial 2 being the highest at 3.85 log_10_ cfu/mL (*P* < 0.01). The average intestinal *C. perfringens* count across all trials was 2.06 log_10_ cfu/mL. These data indicate that day-old chick intestines from various commercial hatcheries (from different locations) have high baseline counts of aerobic and anaerobic microorganisms with the exception of *C. perfringens*.

### Yolk sac microbial counts

The yolk sac counts are shown on Table 3. The yolk sac data demonstrated lower numerical counts for all microorganisms compared to the intestinal data (Table 2). Similar to intestinal data, Trial 6 was excluded from statistical analysis for total aerobes, *E. coli*, and coliforms due to LOD variations (Table 1).

**Table 3:** Average yolk sac microbial counts (log_10_ cfu/mL ± SE) per microorganism evaluated for Trials 1 through 6

Trial	Total aerobes^1^	S. aureus	Gram negatives	E. coli^1^	Coliforms^1^	Yeast and mold	Clostridium spp.	C. perfringens
1	2.13 ± 0.52^b^	1.19 ± 0.36^a^	2.30 ± 0.34^bc^	1.66 ± 0.26^b^	2.40 ± 0.35^b^	3.89 ± 0.08^a^	2.45 ± 0.33^bc^	1.40 ± 0.00
2	4.72 ± 0.77^a^	0.40 ± 0.00^b^	4.32 ± 0.93^ab^	2.75 ± 0.83^b^	4.27 ± 0.92^ab^	3.57 ± 0.29^a^	4.16 ± 0.68^ab^	2.09 ± 0.36
3	3.91 ± 0.49^ab^	0.40 ± 0.00^b^	3.56 ± 0.55^ac^	2.91 ± 0.35^a^	3.35 ± 0.45^ab^	3.62 ± 0.18^a^	3.57 ± 0.48^ab^	1.40 ± 0.00
4	3.61 ± 0.28^a^	2.32 ± 0.55^a^	2.98 ± 0.31^ac^	2.64 ± 0.24^a^	3.07 ± 0.28^ab^	3.22 ± 0.09^a^	4.13 ± 0.34^a^	1.43 ± 0.03
5	4.11 ± 0.64^ab^	1.42 ± 0.71^a^	4.00 ± 0.63^a^	3.51 ± 0.62^a^	4.03 ± 0.60^a^	3.21 ± 0.86^ab^	4.04 ± 0.48^a^	1.40 ± 0.00
6	0.40 ± 0.00	0.40 ± 0.00^b^	2.40 ± 0.00^b^	0.40 ± 0.00	0.40 ± 0.00	2.46 ± 0.04^b^	2.40 ± 0.00^c^	1.40 ± 0.00
Avg 1–5	3.70 ± 0.43	1.15 ± 0.36	3.43 ± 0.36	2.69 ± 0.30	3.43 ± 0.34	3.50 ± 0.13	3.67 ± 0.32	1.54 ± 0.14
Avg 1–6	3.15 ± 0.65	1.02 ± 0.32	3.26 ± 0.34	2.31 ± 0.45	2.92 ± 0.57	3.33 ± 0.20	3.46 ± 0.34	1.52 ± 0.11

^a−c^ Means within a column having different superscripts are significantly different (*P* < 0.05)

^1^ Total aerobes, *E.coli*, and coliform counts in Trial 6 were excluded from statistical analysis due to LOD differences from Trials 1 through 5

The total aerobes and *Clostridium* spp. counts demonstrated low yolk sac microbial densities of 3.70 and 3.67 log_10_ cfu/mL, respectively (excluding Trial 6). For *Clostridium* spp., the hatchery in Trial 6 had the lowest yolk sac *Clostridium* spp. microbial counts of 2.40 log_10_ cfu/mL (*P* < 0.05). Similar to the intestinal results, yolk sac *C. perfringens* counts were minimal throughout all trials, with an overall average of 1.52 log_10_ cfu/mL.

## Discussion

Previous research has indicated that early colonization does occur in the developing embryo and yolk of hatching eggs (Akinyemi et al. 2020; Ding et al. 2017, 2022). In these studies, 16 S rDNA microbiome analyses were used to determine microbial community diversity and identify taxa groups of organisms. Based on these findings, it appears that early embryonic microbial populations can originate from maternal hens, and both environmental factors and host genetic variability can impact both abundance and diversity (Ding et al. 2022). In addition, it has been suggested that the yolk microbiota can lead to increased colonization of certain groups of bacteria in the embryonic intestinal tract (Ding et al. 2017). While previous research has established molecular comparisons of the various microbial populations this does not account for quantitative levels of viable bacteria. Therefore, we enumerated microbial populations on various selective media on day-old chick intestinal tracts and compared these levels to yolk sac content microbial counts. These selective media represented groups of organisms considered important in overall hatching egg viability and poultry health. In the current study, the quantitative levels of selected indigenous microbial populations were generally lower for the yolk sacs versus the day-old intestinal tracts. Previous research reported similar levels for hatching egg intestinal contents for a wide range of selective media including TSA, EMB, and EYA selected microbial populations (Meisinger et al. 2026a). Likewise, lesser populations were detected in eggshell membranes on days 18 to 20 before hatching compared to intestinal populations with most microbial groups increasing at the later dates of hatch (Meisinger et al. 2026b).

Yeast and molds were detected in the current study in both the intestinal and yolk sac contents with lower counts occurring in the yolk sacs. Fungi such as *Aspergillus* and *Fusarium* can produce mycotoxins which can be detrimental to animal productivity (Chavez et al. 2020). Although mycotoxin production is typically associated with fungi present in animal feeds, Wang et al. (2021) demonstrated that residual fungal toxin levels could be detected in breeder hen eggs and eggs inoculated with these same toxins exhibited lower hatching rates and hatched chicks possessed gizzard ulcerations with hemorrhagic lungs. Even though yeasts and molds were detected in both the yolk sacs and the intestinal tracts in the current study, it is not known if these were potential mycotoxin producing fungi and/or if mycotoxins could be generated in the eggs by these fungi. However, Adah et al. (2015), using a different selective media isolated yeasts and molds from dead-in-shell embryos and suggested a potential association with detrimental impacts on hatching egg embryos. A next step would be to demonstrate production of fungal toxins in eggs and follow hatching mortalities and postharvest young chick health and performance.

*Staphylococcus aureus* was detected in much greater levels in the intestinal contents than in the yolk sacs where they were close to the level of detection for this particular selective media. Although a different selective media (mannitol salt agar, MSA) was used in a previous study similar presumptive levels of staphylococci were detected in embryo intestinal contents (Meisinger et al. 2026a). In a different study using this same media, Meisinger et al. (2026b) detected very low staphylococci counts in eggshell membranes but also reported low counts for late hatch intestinal populations as well. In earlier research, Kizerwetter-Świda and Binek (2008) followed the progression of staphylococci from day 18, day 20, and newly hatched chicks in cecal contents, livers, and egg yolk sacs and reported consistent low levels of staphylococci at all stages that were similar to the levels reported here. However, staphylococci were not detected in all of the egg and birds sampled (Kizerwetter-Świda and Binek 2008). It is unclear the impact of staphylococci has on embryo viability, but *S. aureus* has been associated with bacterial chondronecrosis with osteomyelitis (BCO) that causes lameness in birds as young as 14 days (McNamee and Smyth 2000). However, more recent studies have isolated *S. aureus* from dead-in-shell hatching eggs that were resistant to multiple antibiotics and the authors suggested that it could be a causative agent (Khafagy et al. 2025).

Early investigations in poultry by Smith (1965) demonstrated that *E. coli* can be found in day-old chick small intestine at 8.20 and 9.20 log_10_ viable count/g for the anterior and the posterior portions of the intestine, respectively, and at 8.30 log_10_ viable count/g in the ceca. The intestinal *E. coli* microbial levels reported by Smith (1965) were higher than the current study’s average of 6.10 log_10_ cfu/mL (including all trials). While it was not determined in this study the detection of *E. coli* in the yolk sac as well as the intestinal contents could be of concern if some of these isolates are pathogenic. Septicemia from yolk sac infections by avian pathogenic *E. coli* can lead to mortality in young chicks during the first week after hatch (Perez et al. 2025).

The occurrence of certain strains of *C. perfringens* in poultry can be a serious health issue as a causative agent for necrotic enteritis and can also be responsible for foodborne disease in humans (Moore 2016). Early research established a precedent for detecting *C. perfringens* in in young birds. Smith (1965) reported that the day-old chick *C. perfringens* microbial counts for the anterior and posterior portions of the intestine were 4.70 and 5.80 log_10_ viable count/g, respectively, with no detection in the ceca. The current study’s average of all trials for intestinal *C. perfringens* counts (2.06 log_10_ cfu/mL) was lower than reported by Smith (1965). Detection of *C. perfringens* in day old chicks is consistent with the suggestion that intestinal colonization occurs early in the life of the chick (Van Immerseel et al. 2004). Several hatchery sources such as eggshell fragments, chicken fluff and paper pads in the hatchery have been identified as potential transmission agents (Van Immerseel et al. 2004). However, whether the *C. perfringens* in the current study are pathogenic remains to be determined. It is believed that predisposing changes in the intestinal tract microbiota such as inclusion of certain diets may contribute to the proliferation of pathogenic *C. perfringens* (Moore 2016).

More in-depth characterization of these respective microbial isolates is warranted. For example, virulence and antibiotic resistance genetic profiling whether isolates such as *E. coli* or *Staphylococcus* are pathogenic would be important when considering hatchery microbial risks. In addition, identification of organisms by selective plating has limitations as selective media does not always exclude organisms beyond the target organism (Kim et al. 2017; Bodie et al., 2025). Several studies have employed 16 S rDNA microbiome sequencing to establish taxa identification and diversity comparisons between eggs and potential contributors to contamination. However, these results represent proportional estimates of individual microbial taxa and are not quantitative to assess actual microbial loads of the respective contaminant organisms. In addition, this type of data includes all bacteria with no differentiation between viable and nonviable microorganisms. In the future, application of viable microbiome sequencing as well as more precise microbiome sequencing should help to delineate the individual contributions of specific transmission routes for microbial contamination of hatching eggs. Availability of such approaches should help evaluate intervention methods at the hatchery environmental level that are most likely to minimize cross contamination and support gut health in commercial hatching eggs and young chicks.

## Conclusions

Overall, the intestinal microbial densities recovered from the whole digestive tracts of day-old chicks were, on average, higher than those isolated from corresponding yolk sacs. An explanation to these results may be due to the different modes of bacterial transmission during embryonic incubation and subsequent hatching. This would support the hypothesis that yolk sac bound eggshell microbiota slowly traverse the shell pores towards the vitelline membrane during the first days of incubation. Consequently, the total microbial load within the yolk sac remains stable and does not exponentially multiply during the final phase of hatching.

However, as previously suggested by researchers, a different mechanism may drive the development of the intestinal microbiota. The high bacterial counts observed in the chick GIT at hatch suggests that an external environmental source may be the point of contamination. The interaction between the hatchery environment and the intestinal tract of the hatching egg embryo is likely complex with multiple sources for contamination including eggshell and maternal contributions (Maki et al. 2020). This environmental contamination exposes newly hatched chicks and may explain why the intestinal bacterial concentrations are higher than the yolk sac levels. Other hatchery environmental sources may also play a role. Previous work has indicated that many of these same organisms as those found in hatching eggs occur in the hatchery air especially in the last few days of hatch and can be a source of egg contamination (Meisinger et al. 2026b). While the selective culture methods used in the current study have identified organisms that may impact embryo viability, it is difficult to correlate embryonic development contamination to day-old chick microbial loads. Thus, further investigation should be conducted to determine if environmental exposure including airborne microbial loads in the hatcher can be traced more precisely to the gastrointestinal tract microbiota of the chick to become populated.

## Data Availability

Data available from lead author Karely Cantu upon reasonable request.
